# Heterogeneity of aquaporin-4 localization and expression after focal cerebral ischemia underlies differences in white versus grey matter swelling

**DOI:** 10.1186/s40478-015-0239-6

**Published:** 2015-09-30

**Authors:** Jesse A. Stokum, Rupal I. Mehta, Svetlana Ivanova, Edward Yu, Volodymyr Gerzanich, J. Marc Simard

**Affiliations:** Department of Neurosurgery, University of Maryland School of Medicine, 10 S. Pine St., MSTF 634, Baltimore, MD 21201-1595 USA; Departments of Pathology, University of Maryland School of Medicine, 10 S. Pine St., MSTF 634, Baltimore, MD 21201-1595 USA; Department of Physiology, University of Maryland School of Medicine, 10 S. Pine St., MSTF 634, Baltimore, MD 21201-1595 USA

**Keywords:** Ischemic stroke, White matter, Grey matter, Microvessels, Astrocytes, Aquaporin-4

## Abstract

**Introduction:**

Ischemic stroke, a major cause of mortality, is frequently accompanied by life-threatening cerebral edema. Aquaporin-4 (Aqp4), an astrocytic transmembrane water channel, is an important molecular contributor to cerebral edema formation. Past studies of Aqp4 expression and localization after ischemia examined grey matter exclusively. However, as white matter astrocytes differ developmentally, physiologically, and molecularly from grey matter astrocytes, we hypothesized that functionally important regional heterogeneity exists in Aqp4 expression and subcellular localization following cerebral ischemia.

**Results:**

Subcellular localization of Aqp4 was compared between cortical and white matter astrocytes in postmortem specimens of patients with focal ischemic stroke versus controls. Subcellular localization and expression of Aqp4 was examined in rats subjected to experimental stroke. Volumetric analysis was performed on the cortex and white matter of rats subjected to experimental stroke. Following cerebral ischemia, cortical astrocytes exhibited reduced perivascular Aqp4 and unchanged Aqp4 protein abundance. In contrast, white matter astrocytes exhibited increased perivascular and plasmalemmal Aqp4 and a 2.2- to 6.2-fold increase in Aqp4 isoform abundance. Ischemic white matter swelled by approximately 40 %, while cortex swelled by approximately 9 %.

**Conclusions:**

The findings reported here raise the possibility that cerebral white matter may play a heretofore underappreciated role in the formation of cerebral edema following ischemia.

**Electronic supplementary material:**

The online version of this article (doi:10.1186/s40478-015-0239-6) contains supplementary material, which is available to authorized users.

## Introduction

Worldwide, stroke is the second most common cause of death, accounting for approximately 11.5 % of deaths [1]. Ischemic stroke alone accounts for 2.8 million deaths annually [1]. Malignant infarction, characterized by life-threatening cerebral edema, occurs in 1–10 % of patients with supratentorial infarctions, usually within 2–5 days of the ictus, and is associated with a mortality rate of almost 80 % [2–4]. Cerebral edema is also associated with poor patient outcomes, even in the context of non-malignant infarction volumes [5].

In recent decades, knowledge of the mechanisms of cerebral edema formation has greatly improved [6, 7]. Much of this work has focused on cortical or striatal grey matter, partially because rodents, which are commonly used in preclinical studies to model stroke, have relatively little cerebral white matter [8]. However, grey matter and white matter differ in architecture [9–11], cellular composition, and function. There is a growing recognition that the classical morphological division of cerebral astrocytes – key cellular mediators of cerebral edema formation – into white matter (fibrous) and grey matter (protoplasmic) subtypes [12] is developmentally [13–15], molecularly [16–19], and physiologically [20–22] justifiable. Given that the human prefrontal cerebrum is ~2/5ths white matter [23], white matter integrity after stroke is a stronger functional predictor than preserved neurological function [24]. Insofar as certain studies indicate that white matter is highly susceptible to swelling after acute CNS injury [25, 26], it is important to determine whether astrocyte regional heterogeneity manifests as significant differences in certain molecular events that drive cerebral edema formation.

Aquaporin-4 (Aqp4) is a passive transmembrane water channel that in the central nervous system (CNS) is exclusively expressed by astrocytes, and is an important molecular contributor to cerebral edema formation after cerebral ischemia [27–31]. Aqp4 protein abundance is directly correlated with the magnitude of edema [28, 30]. Consensus exists that, in cortical astrocytes, Aqp4 mRNA increases 3 days post-ictus [32, 33]. Protein abundance examined at similar times has been reported to be decreased (~50 %) [34], unchanged [35], or slightly (~10 %) increased [33], with this heterogeneity possibly due to differences in tissue harvesting or to the specific parameters of the stroke model studied.

The subcellular localization of Aqp4 is central to its physiological [36] and pathological [37] roles. In healthy grey and white matter astrocytes, Aqp4 is localized mostly to the perivascular endfeet [38], the terminal pads of large astrocyte processes that completely ensheath all cerebral vessels [10, 39], where it mostly exists as large multimers called orthogonal arrays of intramembraneous particles (OAPs). OAPs cover approximately 50 % of the endfoot plasmalemma [38, 40], where they greatly increase its water permeability [40]. OAP formation is necessary for endfoot localization [41], and is mostly determined by the relative membrane abundance of the two major Aqp4 isoforms, the “small” M23 isoform and the “large” M1 isoform [41–43]. Because the M1 isoform limits OAP size, larger M23:M1 ratios result in larger OAP formation and more Aqp4 localization to the astrocyte endfoot (a.k.a., Aqp4 polarization) [41–43]. The degree of Aqp4 polarization is correlated with the magnitude of edema formation following cerebral ischemia [35, 37, 44, 45]. Following cerebral ischemia, astrocytes alter the M23/M1 expression ratio [46], a transcriptional change that might mechanistically underlie observations that, in grey matter, CNS injury triggers a redistribution of Aqp4 away from the endfoot [35, 47, 48]. This phenomenon has been speculated to protect against local formation of edema fluid [35].

All prior studies of Aqp4 in the context of cerebral ischemia and trauma have focused on grey matter astrocytes. To determine whether white matter and grey matter astrocytes exhibit different patterns of Aqp4 subcellular localization or abundance following cerebral ischemia, here we examined Aqp4 subcellular localization in cortical grey matter versus white matter astrocytes in postmortem brain tissues from humans who had suffered from an ischemic stroke. We also examined Aqp4 subcellular localization and abundance of the M1 and M23 isoforms in cortical grey matter and subcortical white matter (lateral corpus callosum and external capsule) astrocytes at different times after reperfusion in a rat model of middle cerebral artery occlusion (MCAO). To determine whether grey matter and white matter exhibit different propensities for tissue swelling, we compared the relative volume increase exhibited by cortex versus subcortical white matter in the rat MCAO model of stroke.

## Materials and methods

### Human brain tissue

All procedures pertaining to human subjects were approved by the University of Maryland School of Medicine Institutional Review Board. Three patients, 2 men and 1 woman aged 61, 64, and 37 years, who died within 5 days of documented focal cerebral ischemia and who underwent autopsy between January 2010 and December 2012 were retrospectively identified by review of the records of the Department of Pathology. Histological validation of the presence of an ischemic lesion in these cases was made by a board certified neuropathologist (R.I.M.); these tissues were a subset of those used in a previous study [49]. Lesions were located in the right posterior communicating artery territory, the left middle cerebral artery territory, or the right frontal lobe. A 54 year old male and 57 year old female both of whom had died rapidly from acute aortic dissection were used for controls. Postmortem intervals were 15–52 hours. Human brain tissues were submitted to standard postmortem fixation consisting of 7–10 days in formalin and cryoprotection (30 % sucrose) prior to cryosectioning (10 μm) for immunohistochemistry.

### Rat model of stroke

All procedures pertaining to experimental animals were approved by the Institutional Animal Care and Use Committee of the University of Maryland School of Medicine. Male Wistar rats (250 to 300 g; Harlan, Indianapolis, IN) were anesthetized (50 mg/kg ketamine and 7.5 mg/kg xylazine intraperitoneally). Body temperature was maintained with a heating pad regulated by rectal temperature (Harvard Apparatus, Holliston, MA) and rats were allowed to spontaneously ventilate room air. The skull was thinned 2 mm rostral and 2 mm lateral to the bregma, and a laser Doppler probe was affixed to the skull using α-cyanoacrylate adhesive. The right common, external and internal carotid arteries (CCA, ECA, ICA) were exposed by a ventral midline incision. The CCA, distal ECA and pterygopalatine artery were tied off prior to bisection of the ECA. A commercially available intra-arterial occluder (0.39 mm; 4039PK5Re; Doccol Corp, Redlands CA) was used. Middle cerebral artery occlusion (MCAO) was obtained by inserting the occluder retrograde into the external carotid artery (ECA) and advancing it into the ICA under guidance of the Doppler flowmeter. Only animals with a drop in relative cerebral blood flow (rCBF) >75 % were included for further study. After 120 minutes of MCAO, the occluder was withdrawn, the ECA was ligated and flow was restored in the CCA/ICA.

Animals were sacrificed after 2, 10, 24, or 48 hours of reperfusion by intraperitoneal injection of pentobarbital. Uninjured animals served as controls. Following euthanasia, animals were intracardially perfused with saline and 4 % paraformaldehyde. The brain was removed and immersion fixed for 24 hours followed by cryoprotection (30 % sucrose).

To ensure that ketamine anesthesia does not affect Aqp4 expression, total Aqp4 protein abundance was quantified in the cortex and subcortical white matter of animals submitted to intraperitoneal injection of either 50 mg/kg of ketamine (*n* = 3), or an equivalent volume of saline (*n* = 3). Immunoblot revealed no differences in Aqp4 expression in the cortex (*p* = 0.45) or subcortical white matter (*p* = 0.70) between rats administered saline and rats administered ketamine.

### Immunohistochemistry and TUNEL

For human tissues, antigen retrieval was performed prior to immunolabeling. Slides were immersed in IHC-Tek Epitope Retrieval Solution (IHC WORLD, LLC; IW-1100), microwaved for 2 minutes and cooled at room temperature for 3 minutes; this protocol was repeated three times. Sections were allowed to rest at room temperature for 20 minutes, rinsed briefly in phosphate buffered saline (PBS), and blocked for 1 hour in 2 % donkey serum with 0.2 % TritonX-100. Sections were incubated overnight at 4 °C with a primary polyclonal rabbit antibody against Aqp4 (AB3594; Millipore, Billerica, MA) and double labeled with either anti-S100 (ab7852; Abcam, Cambridge, MA) or anti-CD31 (M0823; Dako, Carpinteria, CA) antibody. The following day, Alexa Fluor 550- or fluorescein isothiocyanate-conjugated secondary antibodies (A31570 and A21206; Thermo Fisher Scientific Inc., Waltham, MA) were applied prior to coverslipping with ProLong Gold antifade reagent (P36930; Thermo Fisher Scientific Inc.). Omission of primary antibody was used as a negative control.

For rat tissues, coronal cryosections (12 μm) were obtained starting from approximately 4.5 mm posterior to bregma. Tissues were blocked for 1 hour in 2 % donkey serum and 0.2 % TritonX-100. Sections were then incubated overnight at 4 °C with rabbit primary antibody against Aqp4 (AB3594; Millipore) and double-labeled with mouse primary antibody against GFAP (C9205; Sigma-Aldrich, St. Louis, MO) or rat endothelial cell antigen-1 (RECA-1) (MA1-81510; Thermo Fisher Scientific Inc.). The following day, Alexa Fluor 550- or fluorescein isothiocyanate-conjugated secondary antibodies were applied prior to coverslipping with ProLong Gold antifade reagent (P36930; Thermo Fisher Scientific Inc.) Omission of primary antibody was used as a negative control. Terminal deoxynucleotidyl transferase dUTP nick end labeling (TUNEL) was performed on cryosections as per kit instructions (11684795910; Roche, Branchburg, NJ). See Table 1 for immunohistochemistry markers.

**Table 1:** Marker antigens for immunohistochemistry

Species	Cellular Target	Marker Antigen	Antibody
Human	Endothelial Cells	CD31	M0823; Dako
Human	Astrocytes	S100	ab7852; Abcam
Rat	Endothelial Cells	RECA-1	MA1-81510; Thermo Fisher Scientific Inc.
Rat	Astrocytes	GFAP	C9205; Sigma-Aldrich

The specificity of the anti-Aqp4 antibody was validated by confirmation of expected localization of Aqp4 immunoreactivity in control human and rat cortical tissues and by confirmation of expected molecular weight bands in immunoblot of control rat brain tissue (Additional file 1: Figure. S1).

### Regions of interest - immunohistochemistry

For immunohistochemical analysis of rat brain sections, three mutually exclusive regions of interest (ROI) were defined. The first ROI was defined within the cortical penumbra, a zone of ischemic, but still viable tissue that in the MCAO model reliably includes the superior ACA-MCA watershed region [50–52]. The center of the cortical penumbra ROI was defined as a rectangle of 1 mm width extending from pia to subcortical white matter, set orthogonally to the pial surface and offset laterally from the midline by 3 mm. The value of the lateral offset was obtained from averaging the distance between midline and onset of necrosis in the cortex identified with TUNEL staining among 3 rats sacrificed after 24 hours reperfusion. The second ROI, a square of 500 μm, was placed lateral to the first ROI in the necrotic ischemic core. The third ROI was hand drawn over the subcortical white matter ipsilateral to MCAO to include the lateral corpus callosum and external capsule.

### Image analysis - immunohistochemistry

For quantification of perivascular Aqp4 in rat tissues, sections were double-labeled for Aqp4 and RECA-1 and three epifluorescence images were obtained at random locations within each ROI using a 20x air objective. From each image, a random microvessel oriented parallel to the slice plane was selected. With assistance from the RECA-1 channel, the microvessel was segmented by hand prior to summation of the Aqp4 signal and normalization to the microvessel area. Signals from the three microvessels were then averaged and normalized to the average value of the control microvessels.

### Immunoblot analysis

For quantification of Aqp4 protein abundance in rat tissues, brains from rats submitted to 120 minutes MCAO were harvested after 48 hours reperfusion. Brains were isolated and 3 mm coronal slabs were cut; a slab that bridged from approximately 1 mm anterior to bregma to −2 mm posterior to bregma was isolated. Coronal slabs from control and from rats submitted to MCAO were stained with 2 % triphenyl tetrazolium chloride (TTC) dissolved in 0.9 % saline for 20 minutes at room temperature to determine the location of the ischemic core and penumbra; this technique does not affect the quality of isolated RNA or protein [53]. The necrotic tissue (white) was removed and discarded (the core was not analyzed due to protein degradation) whereas the surrounding cortical penumbra (red) and the ipsilateral subcortical white matter (lateral corpus callosum and external capsule) were dissected, homogenized in lysis buffer (1 % Triton X-100 in 1 x dPBS), and analyzed by immunoblot. Proteins were detected using anti-Aqp4 (AB3594; Millipore) and anti-Hsc-70 (sc-7298; Santa Cruz Biotechnology, Inc., Dallas, TX).

### Silver infarct staining

For silver staining, 12 μm cryosections were briefly washed with PBS, submerged for 2 minutes in a silver impregnation solution with vigorous shaking, washed 6x in deionized water, submerged for 3 minutes in a developer solution, washed 3x in deionized water, and coverslipped with ProLong Gold antifade reagent (P36930; Thermo Fisher Scientific Inc.). The impregnation and the developer solutions were prepared according the protocol described in Vogel et al. [54]. Stained sections were digitized, converted to greyscale and inverted. ROIs used for analysis corresponded to those used for analysis of immunohistochemistry data. The pixel intensity within each ROI was summed and normalized to ROI area.

### Measurement of tissue swelling

For swelling analysis, tissues were obtained from rats submitted to a 4.5 hour MCAO with 24 hours of reperfusion; a longer MCAO was used for this experiment to maximize edema formation. Coronal cryosections of 40 μm thickness separated by 460 μm were sectioned sequentially from a 2 mm coronal block centered at approximately 1 mm posterior to bregma.

First, to distinguish subcortical white matter from cortical grey matter, sections were stained with the myelin stain Black Gold II as per the manufacturer’s instructions (Histo-Chem Inc., USA), and counterstained with Cresyl Violet, a nuclear stain. Stained sections were digitized to the RGB (red-green-blue) color space and converted to the HSI (hue-saturation-intensity) color space, whereupon the hue channel was isolated and used for all downstream image analysis.

For each section, the ipsilateral and contralateral cortical grey matter and subcortical white matter (lateral corpus callosum and external capsule) was segmented for volumetric analysis. To minimize bias in defining the boundary between cortical grey matter and subcortical white matter, the segmentation of subcortical white matter was automated using an active contour segmentation algorithm, as implemented in the *Snake: Active Contour* MATLAB (MathWorks, Natick, MA) toolkit [55]. On each section, an initial contour that outlined the subcortical white matter was initialized by hand. This initial contour was then fitted to the image by optimizing the external and internal energies of the contour through 100 iterations of the active contour algorithm. A medial boundary was then defined on the output subcortical white matter segmentation and corresponded to a line oriented normal to the superior zenith of the rostral-caudal fibers of the corpus callosum. Next, the cortex segmentation was defined by hand; the lateral margin of the subcortical white matter segmentation was used as the medial boundary of the cortical grey matter segmentation. The volume of each segmented region was then estimated by the rectangular estimation of morphometric volume,

$$ V=d{\displaystyle \sum_{i=1}^n{A}_i} $$

where *d* is the distance between sections, *A*_*i*_ is the area of the *ith* of *n* segmented regions, and V is the volume of the segmented region.

### Statistical analysis

Data were analyzed using the R software package (available at www.r-project.org) and plotted using OriginPro version 7 (Origin Lab Corp., Northampton, MA). A value of 0.05 was used to evaluate significance for all tests. For the analysis of immunohistochemistry data, prior to group comparisons with ANOVA and post-hoc tests with Tukey’s test, Levene’s test was applied to ensure homoscedasticity. For immunoblot analysis, Student’s t-test was used for comparison between control and MCAO groups. For measurement of tissue swelling, Student’s t-test was used for comparison.

## Results

### Aqp4 subcellular localization in ischemic stroke

**Fig. 1:**
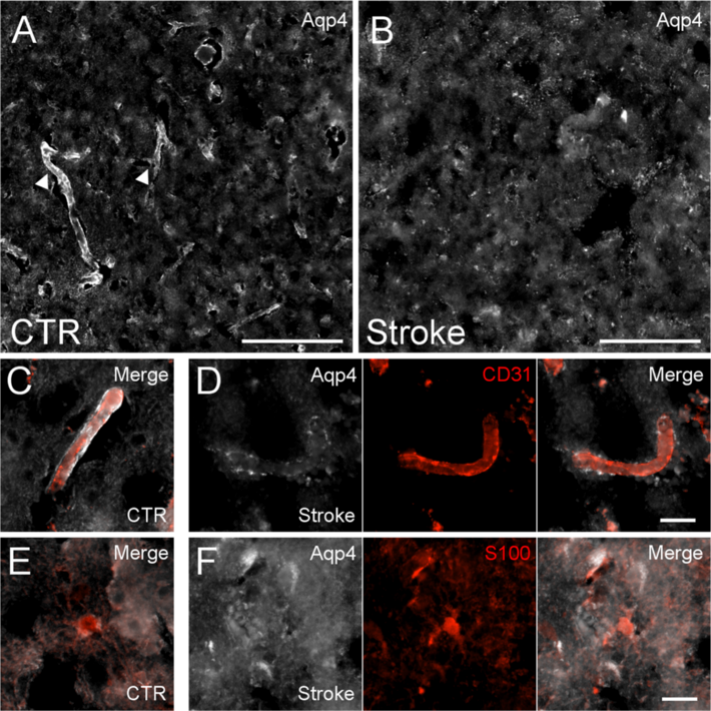
Aqp4 in ischemic human cortex. **a**, **b** montages of micrographs of tissue from control (CTR) human cortex (**a**) or ischemic human cortex from patients with ischemic stroke (**b**) immunolabeled for Aqp4 (*white*), showing perivascular Aqp4 in the control human cortex (filled arrowheads) and loss of perivascular Aqp4 in the ischemic cortex; scale bars 200 μm. **c**-**f** micrographs of control cortical tissue (**c**, **e**) or ischemic cortex (**d**, **f**) immunolabeled for Aqp4 (*white*) and co-labeled for CD31 (*red*) (**c**, **d**) or S100 (*red*) (**e**, **f**), showing attenuation of perivascular Aqp4 in the ischemic cortex (**d**), with no increased Aqp4 in the somata or processes of astrocytes in the ischemic cortex (**f**); micrographs in *C* and *E* depict merged fluorescent channels for Aqp4 and either CD31 or S100; micrographs in *D* and *F* individually depict the Aqp4 channel, the CD31 or S100 channel, and the merged fluorescent image; scale bars 20 μm

Human brain tissues from control and ischemic stroke cases were immunolabeled for Aqp4 to determine whether white matter and grey matter astrocytes exhibit different patterns of Aqp4 subcellular localization following ischemic stroke. In control cortical tissue, cerebral microvessels were strongly immunoreactive for Aqp4, whereas the parenchyma exhibited only weak, diffuse “background” immunoreactivity (Fig. 1a). In contrast, in ischemic grey matter, perivascular Aqp4 immunoreactivity was decreased, indicating loss of Aqp4 polarization (Fig. 1b). Double labeling for CD31, an endothelial marker, confirmed this observation: Microvessels in control cortex exhibited strong and continuous perivascular Aqp4 (Fig. 1c), whereas microvessels in ischemic cortex were outlined by weaker and discontinuous Aqp4 immunoreactivity (Fig. 1d). To determine if Aqp4 was redistributed from the endfoot domain to the plasmalemma of the soma and main processes, tissues were double labeled for S100, an astrocyte marker. Neither astrocytes in the control cortex (Fig. 1e) nor the ischemic cortex (Fig. 1f) exhibited appreciable Aqp4 immunoreactivity at the somata or main processes.

**Fig. 2:**
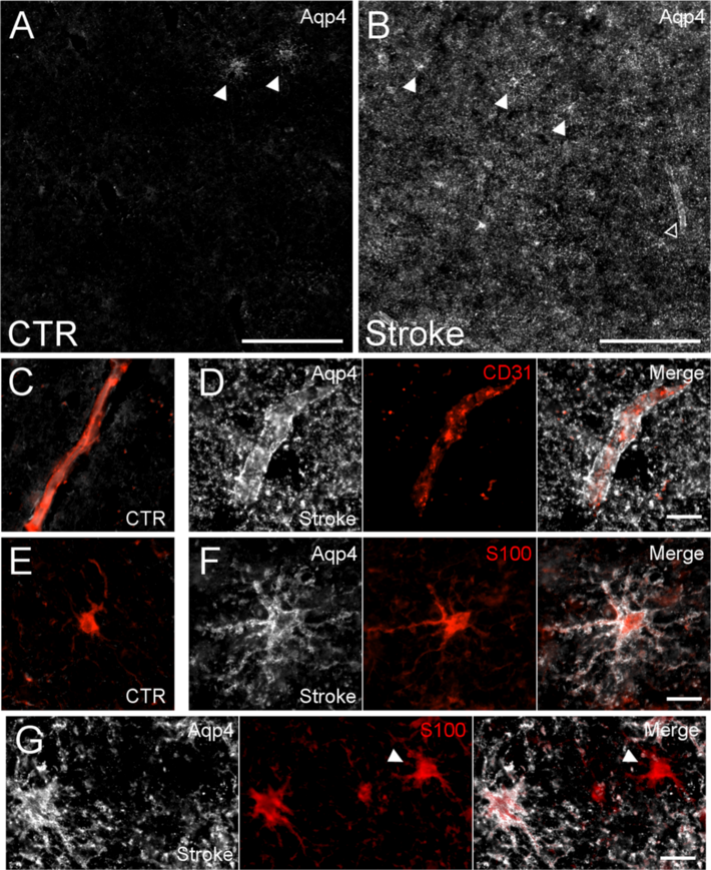
Aqp4 in ischemic human subcortical white matter. **a**, **b** montages of micrographs of tissue from control (CTR) human subcortical white matter (**a**) or ischemic human subcortical white matter from patients with ischemic stroke (**b**) immunolabeled for Aqp4 (*white*), showing minimal Aqp4 expression in control tissue with scarce Aqp4 positive cells (filled arrowheads) and increased Aqp4 immunoreactivity in ischemic white matter in ramified cells (filled arrowheads) and surrounding vessels (empty arrowhead); scale bars 200 μm. (**c**-**g**) micrographs of control white matter (**c**, **e**) or ischemic white matter (**d**, **f**, **g**) immunolabeled for Aqp4 (*white*) and co-labeled for CD31 (*red*) (**c**, **d**) or S100 (*red*) (**e**, **f**, **g**) showing increased perivascular Aqp4 in the ischemic white matter (**d**), increased Aqp4 in the somata and processes of white matter astrocytes in the ischemic white matter (**f**), and rare Aqp4 negative astrocytes in the ischemic white matter (filled arrowhead) (**g**); micrographs in *C* and *E* depict the merged fluorescent channels for Aqp4 and either CD31 or S100; micrographs in *D*, *F,* and *G* individually depict the Aqp4 channel, the CD31 or S100 channel, and the merged fluorescent image; scale bars 20 μm

In control human white matter, the parenchyma was very weakly immunoreactive for Aqp4 (Fig. 2a). In this tissue, infrequent weakly immunoreactive astrocytes were observed; in these cells, Aqp4 was not polarized to the endfoot, but rather was distributed uniformly over the entire plasmalemma (Fig. 2a). Aqp4 immunoreactivity was strikingly increased in ischemic white matter (Fig. 2b). In contrast with microvessels in ischemic grey matter, compared to control (Fig. 2c), microvessels in ischemic white matter exhibited greatly increased perivascular Aqp4 (Fig. 2d). Unlike astrocytes in ischemic grey matter, compared to control (Fig. 2e), astrocytes in ischemic white matter exhibited greatly increased Aqp4 immunoreactivity across the entire plasmalemma (Fig. 2f). Interestingly, in ischemic white matter, rare S100 positive astrocytes remained completely Aqp4 negative (Fig. 2g).

### Rat MCAO model of ischemic stroke

**Fig. 3:**
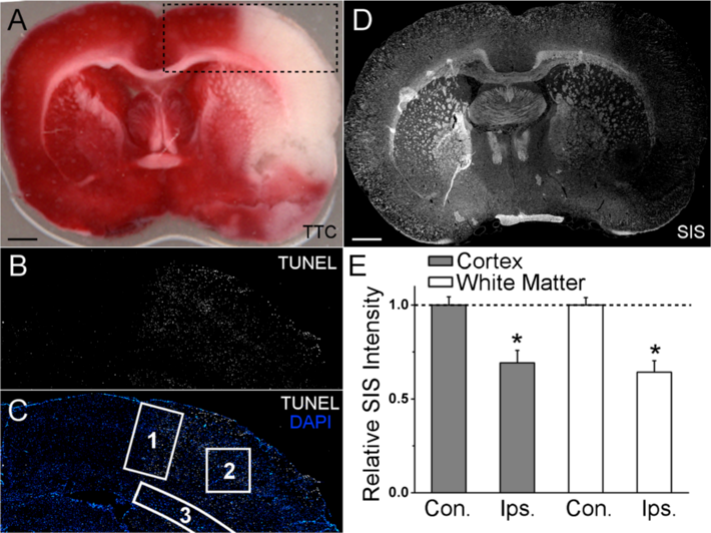
A rat model of ischemic stroke. **a** image of triphenyl tetrazolium chloride (TTC) stained rat brain coronal section, taken through the MCA territory after 2 hour ischemia MCAO with 24 hours of reperfusion showing the extent of the ischemic lesion; scale bar 1 mm. **b** montage of micrographs of rat brain tissue after 2 hour ischemia MCAO and 24 hours of reperfusion processed for terminal deoxynucleotidyl transferase dUTP nick end labeling (TUNEL) (*white*) showing the spread of cell death that ends at penumbral tissue located at the ACA-MCA watershed region; field of view corresponds to the dashed region in *A*. **c** montage of micrographs shown in *B* merged with DAPI channel (*blue*) showing the location of the cortical penumbra ROI (ROI 1), the ischemic core ROI (ROI 2), and the subcortical white matter ROI (ROI 3) used for analysis of immunohistochemistry; field of view corresponds to the dashed region in *A*. **d** montage of micrographs of a coronal section after 2 hour ischemia MCAO and 24 hours of reperfusion processed for silver infarct staining (SIS) showing reduced staining in the ischemic grey matter and ischemic white matter; scale bar 1 mm. **e** visualization of analysis of silver infarct staining in cortex and white matter; Con. = contralateral hemisphere; Ips. = ipsilateral hemisphere; *n* = 3 rats per group; * *p* < 0.05 in comparison to the contralateral baseline, depicted as dotted horizontal line

To better understand the regional heterogeneity of changes in Aqp4 subcellular localization and expression after cerebral ischemia, ischemic stroke was modeled by 2 hour transient rat MCAO with variable reperfusion times. TTC staining indicated that this model results in a cortical and striatal infarct (Fig. 3a). In the transient rat MCAO model, penumbral tissue, i.e. ischemic but still viable tissue, is reliably located at the cortical ACA-MCA watershed [50–52]. The location of the cortical ACA-MCA watershed was estimated by measuring where the spread of necrosis, identified with TUNEL labeling, along the cortex halted at 24 hours reperfusion (Fig. 3b). For analysis of coronal sections from the rat MCAO model, three ROIs were defined: (1) cortical penumbra at the cortical ACA-MCA watershed, (2) necrotic cortical infarct core, located lateral to the cortical penumbra ROI and (3) the ipsilateral subcortical white matter including the lateral corpus callosum and external capsule (Fig. 3c).

Given that the subcortical white matter was TUNEL negative (Fig. 3b), we sought to confirm that the ipsilateral subcortical white matter was indeed submitted to ischemic injury in the rat MCAO model. As TTC is a poor indicator of white matter ischemia, we instead used silver staining, a protocol that is sensitive to post-ischemic changes in both grey matter and white matter [54]. In tissue obtained from rats after 24 hours of reperfusion, silver staining was attenuated in a spatial pattern (Fig. 3d) that closely corresponded to the ischemic region identified with TTC (Fig. 3a). Quantification revealed that, compared with contralateral cortex and subcortical white matter, silver staining of the ipsilateral cortex was decreased by 30.8 % ± 4.37 % (*p* = 0.003) and silver staining of the ipsilateral subcortical white matter was decreased by a nearly equivalent 35.8 % ± 6.08 % (*p* = 0.002) (Fig. 3e). Absence of TUNEL labeling, coupled with attenuated silver staining indicated that the ipsilateral subcortical white matter was submitted to ischemic injury, but ultimately remained viable and non-necrotic.

### Aqp4 immunoreactivity after MCAO

**Fig. 4:**
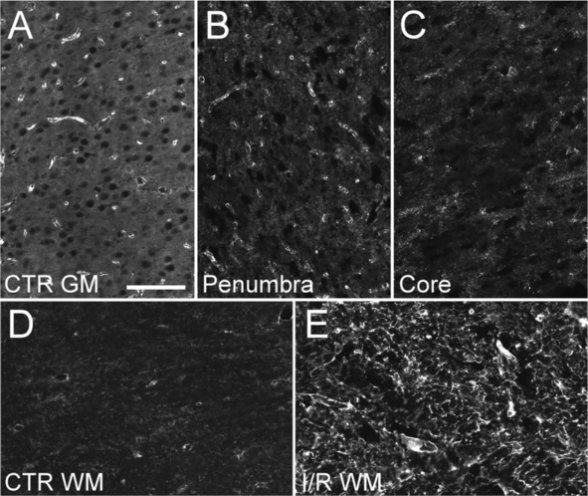
Aqp4 subcellular distribution after MCAO. **a**-**e** montages of micrographs of rat brain tissue immunolabeled for Aqp4 (*white*) in the control (CTR) cortical grey matter (GM) (**a**), the cortical penumbra after 2 hour ischemia and 24 hours reperfusion (**b**), the cortical infarct core after 2 hour ischemia and 24 hours reperfusion (**c**), the control subcortical white matter (WM), including the lateral corpus callosum and external capsule (**d**), or the ipsilateral subcortical white matter 2 hours ischemia and 48 hours reperfusion (I/R) (**e**) showing decreased perivascular Aqp4 in the cortical penumbra and cortical infarct but increased Aqp4 in the subcortical white matter after ischemia; scale bar 100 μm

In the control cortex, the vasculature was outlined by strong and continuous Aqp4 immunoreactivity and the parenchyma was weakly immunoreactive (Fig. 4a). Perivascular Aqp4 immunoreactivity was weaker in the cortical penumbra after 24 hours of reperfusion (Fig. 4b) and largely absent in the cortical infarct core after 24 hours of reperfusion (Fig. 4c).

In the control subcortical white matter, microvessels exhibited moderate Aqp4 immunoreactivity and the parenchymal labeling was weak in intensity and reticulate in appearance (Fig. 4d). Aqp4 immunoreactivity greatly increased in subcortical white matter after 48 hours of reperfusion and appeared to outline cellular processes and cerebral microvessels (Fig. 4e), a pattern that differed markedly from that observed in the ischemic cortex.

### Perivascular Aqp4 after MCAO

We sought to determine if regionally heterogeneous changes occur in Aqp4 localization to the perivascular endfoot. Tissues from rats subjected to 120 minute MCAO with 2, 10, 24, or 48 hours of reperfusion were double-labeled for RECA-1, a marker for rat endothelial cells, and analyzed for perivascular Aqp4 intensity.

**Fig. 5:**
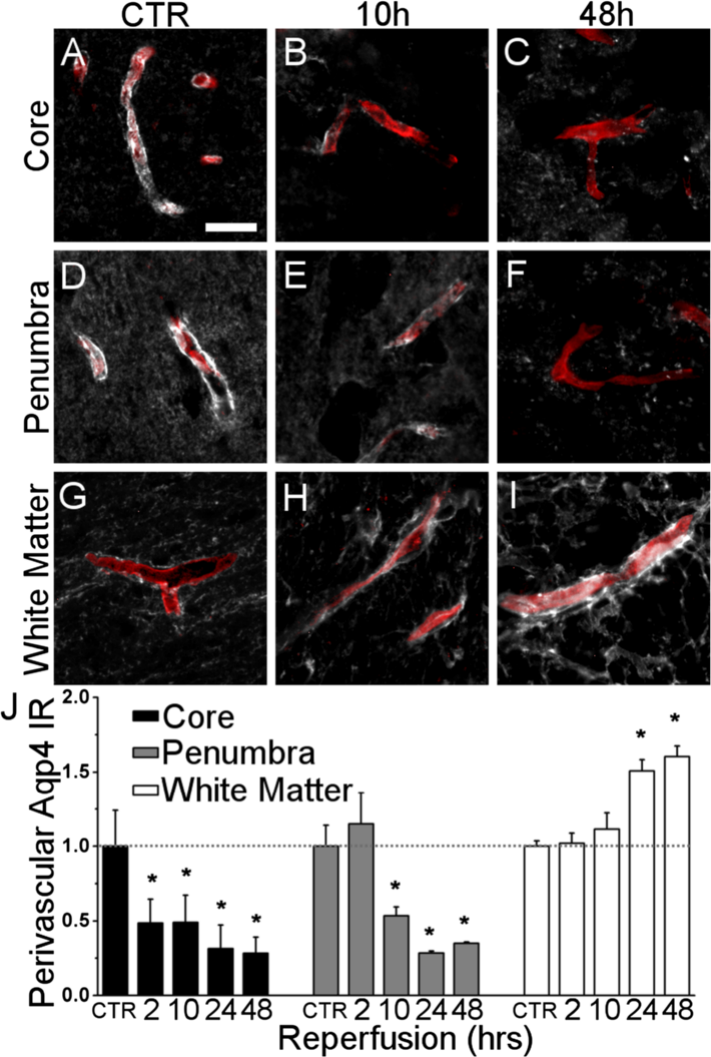
Perivascular Aqp4 after MCAO. **a**-**i** micrographs of rat brain tissue immunolabeled for Aqp4 (*white*) and co-labeled for RECA-1 (*red*) in the control (CTR) cortex (**a**, **d**), the control subcortical white matter (**g**), the cortical infarct core at 10 and 48 hours reperfusion (**b**, **c**), the cortical penumbra at 10 and 48 hours reperfusion (**e**, **f**), or the subcortical white matter ipsilateral to MCAO at 10 and 48 hours reperfusion (**h**, **i**) showing loss of perivascular Aqp4 in the cortical infarct core by 10 hours reperfusion, attenuated perivascular Aqp4 in the cortical penumbra by 10 hours reperfusion, and increased perivascular Aqp4 in the subcortical white matter by 48 hours reperfusion. **j** visualization of temporal analysis of perivascular Aqp4 in experimental ROIs; IR = immunoreactivity; abscissa denotes control animals (CTR) and reperfusion-time after 2 hour ischemia; *n* = 4 rats per group; * *p* < 0.05 in comparison to the control baseline, depicted as dotted horizontal line

In control cortical tissue, microvessels were outlined by strong Aqp4 immunoreactivity (Fig. 5a, d). In the cortical infarct core, perivascular Aqp4 was nearly absent after 10 hours of reperfusion (Fig. 5b) and remained weakly immunoreactive after 48 hours of reperfusion (Fig. 5c). Similar, but temporally delayed changes in perivascular Aqp4 were observed in the cortical penumbra: Here, perivascular Aqp4 became attenuated after 10 hours of reperfusion (Fig. 5e) and was largely absent by 48 hours of reperfusion (Fig. 5f).

In control subcortical white matter, perivascular Aqp4 immunoreactivity was visibly weaker than that in the control cortex (Fig. 5g). After 10 hours reperfusion, microvessels in the subcortical white matter remained outlined by weak Aqp4 immunoreactivity (Fig. 5h). However, after 48 hours reperfusion, perivascular Aqp4 immunoreactivity in the subcortical white matter strongly increased to levels similar to or greater than those observed in the control cortex (Fig. 5i).

The temporal pattern of perivascular Aqp4 immunoreactivity was quantified in the three ROIs. After homoscedasticity was confirmed with the Levene test in the cortical penumbra (W = 0.7095 *p* = 0.598), cortical infarct core (W = 0.2207, *p* = 0.9227), and subcortical white matter (W = 0.3058 *p* = 0.8676), ANOVA revealed that significant differences existed among experimental groups in the penumbra (F = 17.94, p = 1.38x10^−5^), cortical infarct core (F = 13.43, *p* = 7.57x10^−5^) and subcortical white matter (F = 21.52, *p* = 6.69x10^−5^). Post-hoc tests revealed that perivascular Aqp4 intensity in penumbra was lower than control levels at 10, 24 and 48 hours reperfusion, that perivascular Aqp4 intensity in cortical infarct core was lower than control levels at 2, 10, 24, and 48 hours reperfusion, and that perivascular Aqp4 immunoreactivity in ischemic subcortical white matter was higher than control levels at 24 and 48 hours reperfusion (Fig. 5j).

### Aqp4 redistribution after MCAO

We next sought to determine if regionally heterogeneous changes occur as to where Aqp4 is redistributed on the greater astrocyte plasmalemma. Tissues from rats submitted to 120 minute MCAO with 2, 10, 24, or 48 hours of reperfusion were double-labeled for GFAP, the astrocyte-specific intermediate filament. Notably, GFAP labeling was quickly lost in the cortical infarct core, a change that likely reflected astrocyte cell death and tissue necrosis. Therefore, the present analysis was restricted to the cortical penumbra and subcortical white matter, where GFAP labeling was not lost following MCAO.

**Fig. 6:**
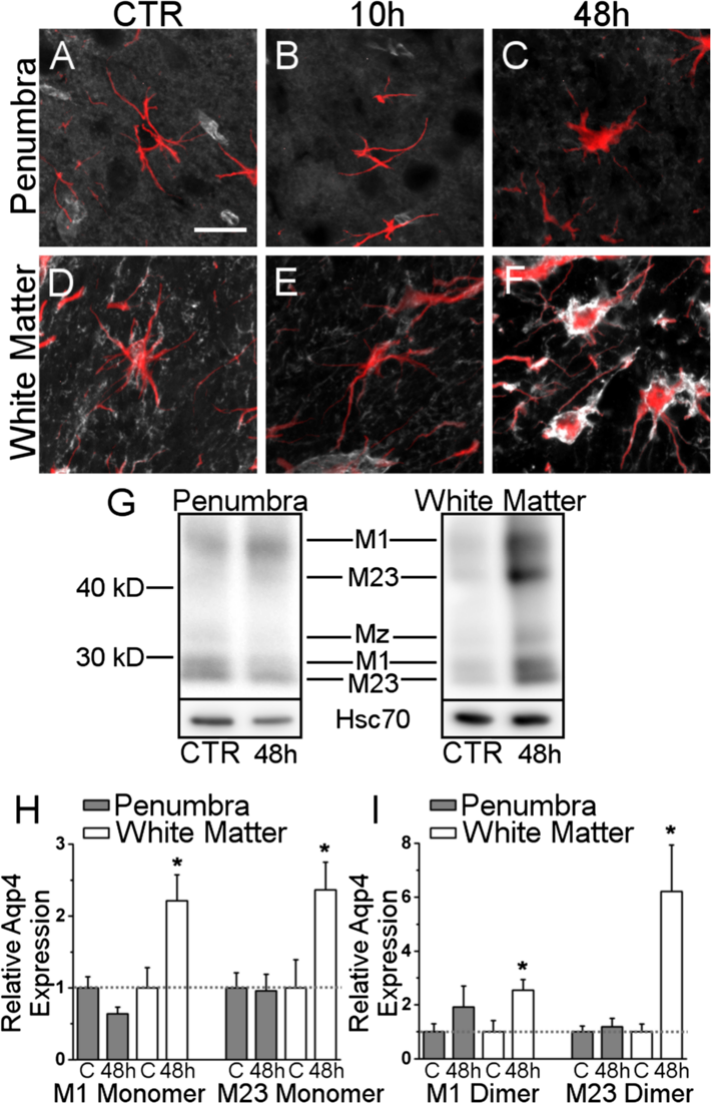
Soma plasmalemma Aqp4 and Aqp4 abundance after MCAO. **a**-**f** micrographs of rat brain tissue immunolabeled for Aqp4 (*white*) and co-labeled for GFAP (*red*) in the control (CTR) cortex (**a**), the control subcortical white matter (**d**), the cortical penumbra at 10 and 48 hours reperfusion (**b**, **c**), or the subcortical white matter ipsilateral to MCAO at 10 and 48 hours reperfusion (**e**, **f**) showing low Aqp4 immunoreactivity at the astrocyte soma plasmalemma in the cortical penumbra at 48 hours reperfusion and increased Aqp4 at the astrocyte soma plasmalemma in the subcortical white matter at 48 hours reperfusion. **g** Aqp4 immunoblot of control tissue and cortical penumbra and subcortical white matter following 2 hour ischemia and 48 hours of reperfusion; 48 h = tissue obtained from rats submitted to 120 minute MCAO and 48 hours of reperfusion. **h**, **i**) visualization of quantification of immunoblot in *G* for Aqp4 monomer isoforms (**h**) and Aqp4 dimer isoforms (**i**); the ordinate reflects Aqp4 isoform band optical density normalized to Hsc-70 band optical density and then normalized to control tissue baseline; C = control tissue; *n* = 4 rats per group; * *p* < 0.05 comparison to the control baseline, depicted as dotted horizontal line

In the control cortex, the plasmalemmae of astrocytic somata and non-endfoot processes were only weakly Aqp4 immunoreactive (Fig. 6a). At no time examined after reperfusion did these cellular structures display increased Aqp4 immunoreactivity, although penumbral astrocytes after 48 hours of reperfusion were hypertrophied and exhibited increased GFAP immunoreactivity, both indications of a reactive phenotype (Fig. 6b, c).

In control subcortical white matter, the astrocyte somata and processes exhibited moderate Aqp4 immunoreactivity (Fig. 6d), a pattern that was largely unchanged after 10 hours of reperfusion (Fig. 6e). However, after 48 hours of reperfusion, subcortical white matter astrocytes exhibited greatly increased Aqp4 immunoreactivity at the somata and main processes (Fig. 6f), a pattern that was appreciably different than the pattern exhibited by astrocytes in the cortex. Like astrocytes in the cortical penumbra after 48 hours of reperfusion, subcortical white matter astrocytes after 48 hours of reperfusion were hypertrophied and exhibited increased GFAP immunoreactivity (Fig. 6f).

### Aqp4 abundance after MCAO

We sought to determine if regionally heterogeneous changes occur in Aqp4 abundance. The cortical penumbra and the ipsilateral subcortical white matter were analyzed with immunoblot. Immunoblot resulted in two bands at ~30 kDa, a band at ~33 kDa, and two bands at ~40-50 kDa, which correspond to the expected molecular weights for monomeric M1, M23, monomeric Mz, and dimeric M1 and M23, respectively [56].

After 48 hours of reperfusion, the cortical penumbra did not exhibit altered abundance of monomeric M1, M23, or Mz, or dimeric M1 or M23 (Fig. 6g). In contrast, after MCAO, subcortical white matter exhibited increased abundance of all isoforms of Aqp4 (Fig. 6g).

Quantification of the abundance of Aqp4 monomers in cortical penumbra confirmed unchanged abundance of monomeric M1 or M23 after MCAO (Fig. 6h). In contrast, quantification of the abundance of Aqp4 monomers in subcortical white matter revealed a 2.2 ± 0.36–fold increase (*p* = 0.024) in monomeric M1 and a 2.4 ± 0.38–fold increase (*p* = 0.028) in monomeric M23 after MCAO compared to control (Fig. 6h). Quantification of the monomeric Mz isoform revealed that in cortical grey matter, monomeric Mz abundance was unchanged after MCAO, while subcortical white matter exhibited a 2.2 ± 0.26–fold increase (*p* = 0.03) in monomeric Mz after MCAO compared to control.

Quantification of the abundance of Aqp4 dimers in cortical penumbra confirmed unchanged abundance of dimeric M1 or M23 after MCAO, although the M1 dimer trended towards greater abundance after MCAO (Fig. 6i). In contrast, quantification of the abundance of Aqp4 dimers in subcortical white matter revealed a 2.5 ± 0.39–fold increase (*p* = 0.021) in dimeric M1 and a 6.2 ± 1.72–fold increase (*p* = 0.038) in dimeric M23 after MCAO compared to control (Fig. 6i).

### Regional swelling after MCAO

Since Aqp4 has been associated with astrocyte and tissue swelling, we sought to determine if cortical grey matter and subcortical white matter swell with different relative magnitudes following MCAO. Coronal slices were stained with Black Gold II, a myelin stain, and counter stained with cresyl violet, a nuclear stain, prior to digitization, segmentation, and volumetric quantification of ipsilateral versus contralateral cortex and subcortical white matter.

**Fig. 7:**
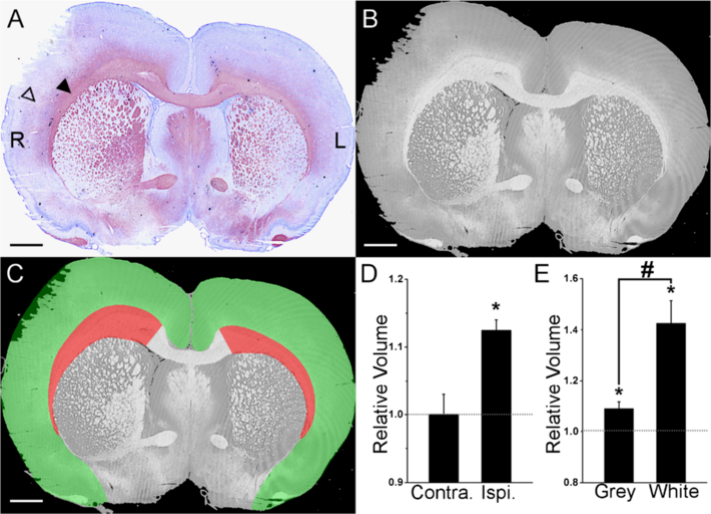
Regional swelling after MCAO. **a** image of a coronal slice of brain tissue from a rat submitted to 4.5 hour ischemia and 24 hours of reperfusion, stained with the myelin stain Black Gold II and counterstained with cresyl violet, demonstrating the raw image data prior to volumetric analysis; lateral corpus callosum (filled arrowhead), cortex (empty arrowhead). **b** hue channel obtained from red-green-blue to hue-saturation-intensity transformation of *A*, demonstrating the input image to the algorithm used for automated segmentation of subcortical white matter. **c** image of output from active contour segmentation of *B* showing ipsilateral and contralateral cortex segmentations (green) and subcortical white matter segmentations (red) overlaid with the input hue image in *B*. **d** visualization of quantification of hemispheric swelling; the ordinate reflects the combination of the ipsilateral (Ipsi.) or contralateral (Contra.) segmentations normalized to the combination of the contralateral segmentations; *n* = 5 rats per group; * *p* < 0.05 comparison to the contralateral baseline, depicted as dotted horizontal line. **e** visualization of quantification of swelling of cortex (Grey) and subcortical white matter (White); the ordinate reflects the volume of either the ipsilateral cortical grey matter or ipsilateral subcortical white matter normalized to the volume of the contralateral cortex or subcortical white matter; *n* = 5 rats per group; * *p* < 0.05 comparison to the contralateral baseline, depicted as dotted horizontal line; # *p* < 0.05 comparison between cortical grey matter versus subcortical white matter

Stained coronal sections exhibited intense Black Gold II staining in the subcortical white matter (corpus callosum and external capsule) and strong cresyl violet staining in the cerebral cortex, with these regions separated by an indistinct boundary (Fig. 7a). The right (ipsilateral) side exhibited swelling in both cortex and subcortical white matter with apparent necrosis in the superior-lateral cortex (Fig. 7a). Input images were transformed from RGB to HSI color space, whereupon the hue channel was isolated (Fig. 7b). Segmentation of each hue image yielded 4 ROIs, corresponding to ipsilateral and contralateral cortex and subcortical white matter (Fig. 7c). The area occupied by each segmented ROI was calculated and, using the rectangular estimation of morphometric volume, was used to calculate the volume of each ROI.

The ipsilateral and contralateral hemispheric volume, excluding the striatum and diencephalon, were estimated as the combined ipsilateral ROIs (cortex plus subcortical white matter) and the combined contralateral ROIs. Overall, the ipsilateral hemisphere was 12.5 % ± 3 % larger in volume than the contralateral hemisphere (*p* = 0.006) (Fig. 7d).

Next, we sought to determine the relative volume increase exhibited by the cortex and the subcortical white matter. The ipsilateral cortex was 13.23 ± 3.95 mm^3^ larger in volume than the contralateral cortex (*p* = 0.02), an 8.95 % ± 2.64 % increase (Fig. 7e). A similar calculation revealed that the ipsilateral subcortical white matter was 7.42 ± 1.73 mm^3^ larger in volume (*p* = 0.017) than the contralateral subcortical white matter, a 42.5 % ± 8.84 % increase (Fig. 7e). Importantly, the relative increase in volume exhibited by subcortical white matter was significantly larger than that exhibited by cortical grey matter (*p* = 0.011) (Fig. 7e).

## Discussion

In the present study, we demonstrated that subcortical white matter and cortical grey matter astrocytes react to ischemia with strikingly different changes in Aqp4 subcellular localization and expression, and that subcortical white matter is much more susceptible to post-ischemic tissue swelling than cortical grey matter. The findings reported here have important implications for the field of astrocyte regional heterogeneity [22], and for the current understanding of the cellular and molecular mechanisms that drive cerebral edema formation and clearance.

The present study is the first to examine post-ischemic changes in Aqp4 expression or localization in cerebral white matter, and is the first to contrast these changes with those that occur in grey matter astrocytes; all other studies of Aqp4 in the context of cerebral ischemia have focused on grey matter [32–34, 46, 47, 57–59]. The present study also is novel in that it is the first to report molecular differences between reactive white matter and grey matter astrocytes in the ischemic brain; prior studies of regional heterogeneity in reactive cerebral astrocytes have either focused on grey matter [60, 61], or compared cell survival after ischemia [62, 63].

In rat and human cortical astrocytes, perivascular Aqp4 was quickly attenuated following cerebral ischemia, which, in the rat cortical penumbra, was accompanied by unchanged Aqp4 abundance of either the M1 or M23 isoform in either the monomeric or dimeric state. This post-ischemic pattern of subcellular localization and protein abundance is similar to that reported elsewhere [34, 35, 48, 58], and has been interpreted as a redistribution of Aqp4 away from the astrocyte endfoot. The mechanism underlying this redistribution is unclear: While the M1 homodimer, the isoform that tends to reduce OAP size and Aqp4 polarization [41, 46], trended upwards in cortical astrocytes, the M23:M1 ratio was largely unchanged after ischemia. This result indicates that a decreased M23/M1 ratio is not a necessary prerequisite for Aqp4 plasmalemmal redistribution to occur. Alternatively, loss of Aqp4 polarization might have been driven by degradation of vascular basement membrane proteins, some of which function to anchor Aqp4 at the endfoot [64–67].

Surprisingly, in cortical astrocytes, we did not observe increased Aqp4 immunoreactivity at the astrocytic somata or main processes; this phenomenon was previously reported to co-occur with reduced perivascular localization [47]. This discrepancy with the prior literature may be due to differences in the species analyzed (human and rat versus mouse), details of the stroke model (120 min rat MCAO versus 30 min mouse MCAO), or regional heterogeneity between cortical and striatal astrocytes [68], as the latter were examined in the aforementioned study. While the final location of Aqp4 in astrocytes in the ischemic cortex is unclear, the diffuse pattern of immunoreactivity in the parenchyma of the cortical penumbra may suggest that Aqp4 is localized to the finely ramified cortical astrocyte processes, perhaps to participate in glutamate/water buffering [69].

We found that, in contrast with astrocytes in the ischemic cortex, astrocytes in ischemic subcortical white matter exhibited *increased* perivascular endfoot Aqp4 following ischemia. Increased endfoot Aqp4 may have been driven by the increased dimeric M23, and hence the increased M23/M1 ratio observed in immunoblot analysis. Notably, interpretation of the M23/M1 ratio and the dimer state of Aqp4 [56] is difficult, as scant literature exists regarding these topics. Regarding the former, one study reported that the M1 isoform, but not M23 isoform, was increased in striatal astrocytes [46].

Furthermore, and again in contrast with astrocytes in the ischemic cortex, astrocytes in ischemic subcortical white matter exhibited a 2.2–6.2 fold increase in Aqp4 protein abundance, and greatly upregulated Aqp4 at the plasmalemma of the soma and main processes. The overall magnitude of Aqp4 upregulation in subcortical white matter was visually striking, and is best appreciated in Fig. 2a, b and Fig. 4d, e.

Interestingly, we found that cortical grey matter and subcortical white matter exhibit greatly different propensities for tissue swelling after cerebral ischemia. After ischemia, the ipsilateral cortical grey matter was swollen by 8.95 % ± 2.64 % compared to the contralateral cortex, while the ipsilateral subcortical white matter was swollen by a remarkable 42.5 % ± 8.84 %.These findings are in alignment with data from prior studies showing that white matter is highly susceptible to swelling after acute CNS injury [25, 26]. There are at least two possible mechanistic explanations for the striking swelling exhibited by subcortical white matter: firstly, for reasons yet to be clarified, subcortical white matter may be more susceptible to the formation of edema fluid than cortical grey matter; secondly, subcortical white matter might be simply serving as a sink for edema fluid generated elsewhere [70]. Notably, given that the relative volume of white matter to total brain volume is lower in rodents compared with humans [71], these data cannot be directly extrapolated to human stroke.

While the data contained in the present study do not establish a causal link between the described molecular and morphological changes, it is likely that these phenomena are connected. The changes in Aqp4 subcellular localization observed in cortical grey matter astrocytes have been linked with reduced edema formation: Loss of Aqp4 polarization has been speculated to protect against edema formation [35], as mice that lack Aqp4 scaffolding proteins exhibit minimal perivascular Aqp4 and are protected against brain swelling [35, 37, 44, 45]. Thus, we postulate that the reduction of Aqp4 polarization observed to occur in cortical astrocytes partially protected the ischemic cortex from local formation of cerebral edema. In contrast, the changes in Aqp4 expression and subcellular localization observed in subcortical white matter astrocytes, namely, Aqp4 upregulation and greater Aqp4 polarization, are associated with worsened cytotoxic edema, greater local formation of ionic edema and more severe brain swelling [28, 30]. Thus, we postulate that upregulation of Aqp4 expression in subcortical white matter astrocytes, coupled with increased Aqp4 expression at the perivascular endfeet of subcortical white matter astrocytes, facilitated local formation of cerebral edema and hence worsened tissue swelling in the subcortical white matter. With regards to future work, while causality might be established indirectly through a detailed temporal analysis of Aqp4 expression and swelling, perhaps in the 4.5 hour ischemia rat MCAO model, direct evidence for causality requires the development of techniques that might allow for the selective manipulation of Aqp4 expression in white matter astrocytes.

Overall, this study raises the intriguing possibility that white matter may play a heretofore underappreciated *active* role in the formation of cerebral edema following ischemia. Previously, it was assumed that cerebral edema formation occurs mostly in grey matter due to its relatively high vascular density [11]; in contrast, white matter was thought to serve as a mere sink [72] and/or conduit [70, 73] for the accumulation or spread of cerebral edema. However, our findings suggest that, following ischemia, white matter astrocytes exhibit unique molecular changes that may directly facilitate local formation of cerebral edema.

The present study has potential limitations. Firstly, our experimental group underwent surgery under ketamine anesthesia, whereas our controls did not have anesthesia. Certain anesthetic agents can influence Aqp4 localization [74] and vascular tone [75]. However, in the present study, ketamine has been shown not to influence Aqp4 expression in rats. Furthermore, in a previous study, ketamine was shown not to influence Aqp4 expression or edema formation in rats submitted to MCAO [76]. Secondly, given that a direct marker of astrocyte viability was not included, it is possible that astrocyte cell death underlies some of the reported changes in Aqp4 immunoreactivity in the ischemic penumbra. However, this is unlikely since penumbral astrocytes exhibited preserved GFAP immunoreactivity, which correlates strongly with viability [77].

The ischemic threshold for white matter (~20 ml∙100 g^-1^∙min^−1^) is lower than that for grey matter (~35 ml∙100 g^−1^∙min^−1^) [78], a difference that might have resulted in the TUNEL-positive ipsilateral cortex and striatum, but TUNEL-negative ipsilateral subcortical white matter observed in the rat MCAO model (Fig. 3b). Thus, we sought to confirm that the rat white matter examined in the present study was ischemic and damaged. As the commonly used TTC staining technique is not appropriate for identification of ischemic white matter, we utilized an alternative method, silver infarct staining, where the staining intensity is proportional to tissue perfusion [54]. We found that, in rats submitted to MCAO, silver staining was reduced by approximately 30 % in both ipsilateral white matter and grey matter. These findings, which are consistent with past work demonstrating that white matter is highly sensitive to ischemia [79], indicate that the subcortical white matter was indeed submitted to ischemic injury, although it remained viable, analogous to the cortical penumbra.

Following acute CNS injury, astrocytes transition into a so-called “reactive” phenotype characterized by altered gene expression, morphology, and cytokine secretion [80]. While cerebral white and grey matter astrocytes are known to exhibit developmental [13, 14], molecular [16, 17], and physiological [21, 22] differences, prior to the present study, it was unknown if their reactive phenotypes exhibit molecular differences. If the results of the present study, which examined a single protein, extend to other astrocytic proteins, white matter and grey matter astrocyte subtypes may engage fundamentally different programs of reactivity following injury. Given the proportionally large volume of white matter in the human brain [23], this concept could have important implications for a number of astrocyte-mediated phenomena in the injured brain.

## Conclusions

In summary, we report that unlike cortical grey matter astrocytes, which exhibit decreased perivascular Aqp4 and unchanged Aqp4 abundance following ischemia, subcortical white matter astrocytes exhibit greatly increased perivascular Aqp4, plasmalemmal Aqp4, and Aqp4 abundance; this molecular heterogeneity may underlie the relatively greater propensity for tissue swelling exhibited by subcortical white matter. Our findings may have important implications for the formation of cerebral edema and brain swelling following cerebral ischemic injury.

## Additional file

Additional file 1:Figure S1. Validation of Aqp4 antibody. (PDF 1092 kb)
