# Supplementary material for: Heterogeneity of aquaporin-4 localization and expression after focal cerebral ischemia underlies differences in white versus grey matter swelling
Source: Acta Neuropathol Commun. 2015 Sep 30;3:61. doi: 10.1186/s40478-015-0239-6 (PMC4588314; doi:10.1186/s40478-015-0239-6)
Supplement: Additional file 1:Figure S1. — Validation of Aqp4 antibody. (PDF 1092 kb) [file 40478_2015_239_MOESM1_ESM.pdf]

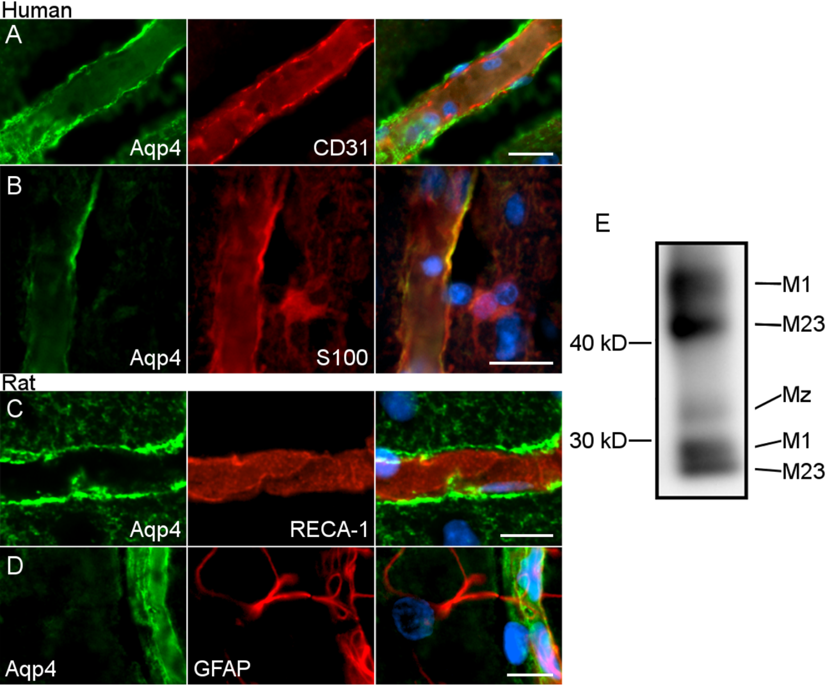

SUPPLEMENTAL FIGURE 1 Validation of Aqp4 antibody. (**A, B**) micrographs of human control brain tissue immunolabeled for Aqp4 (green) and co-labeled for CD-31 (red) or S100 (red). (**C, D**) micrographs of rat control brain tissue immunolabeled for Aqp4 (green) and co-labeled for RECA-1 (red) or GFAP (red); co-labeling is indicated by yellow hue; Aqp4 immunoreactivity was present surrounding cerebral vessels in human tissue (**A**) and rat tissue (**C**), and was localized to astrocyte endfeet, but not astrocyte somata in human tissue (**B**) and in rat tissue (**D**). Scale bar 20 μm. (**E**) Aqp4 immunoblot showing M23 and M1 Aqp4 isoforms in monomer form at ~26-29 kDa and in dimer form at ~40-50 kDa, and Mz Aqp4 isoform at ~33 kDa, demonstrating specificity of anti-Aqp4 antibody
